# Predictors of stages of adoption of colorectal cancer screening among adults attending primary healthcare centers in Turkey

**DOI:** 10.1186/s42506-025-00185-z

**Published:** 2025-03-04

**Authors:** Elif Dönmez, Nadire Ercan Toptaner, Elvan E. Ata, Zeynep Dülger, Onur Acar

**Affiliations:** 1 Oncology Nursing Department, Hamidiye Faculty of Nursing, University of Health Sciences, Istanbul, Turkey; 2 Nursing Department, Faculty of Health Sciences, Istanbul Galata University, Istanbul, Turkey; 3 Psychiatric Nursing Department, Hamidiye Faculty of Nursing, University of Health Sciences, Istanbul, Turkey; 4 Chemotherapy Department, Avrasya Hospital, Istanbul, Turkey; 5 Bursa Orhangazi District Health Directorate, Ministry of Health, Bursa, Turkey

**Keywords:** Colonoscopy, Predictors, Screening, Fecal occult blood, Primary care, Colorectal cancer, Stages of change

## Abstract

**Background:**

Despite the proven effectiveness of colorectal cancer (CRC) screening in reducing mortality, adherence rates for fecal occult blood testing (FOBT) and colonoscopy remain low among Turkish adults. This study aimed to assess the stages of adoption of CRC screening behaviors, identify the factors influencing adoption, and examine the perceived benefits and barriers to screening.

**Methods:**

A cross-sectional study was conducted in Istanbul’s Anatolian region from May to June 2022, involving 498 adults aged 50–70 years. Multistage cluster sampling was used to select 20 primary healthcare centers. A structured questionnaire was administered to assess sociodemographic characteristics, health perceptions, knowledge of colorectal cancer (CRC) screening, and readiness for screening based on the transtheoretical model. The benefits and barriers to screening were evaluated using the Turkish version of “The Instruments to Measure CRC Screening Benefits and Barriers.”

**Results:**

The participants (mean age: 59.10 ± 5.71 years) showed greater recognition of colonoscopy (68.7%) than FOBT (39.8%). Most participants were in the precontemplation stage for FOBT (58.6%) and colonoscopy (63.9%). Perceptions of benefits were associated with education, employment, income, health status, familial CRC history, and screening knowledge (*p* < 0.05). Perceived barriers were linked to lack of knowledge and provider recommendations (*p* < 0.05). The participants in the precontemplation stage reported fewer benefits and more barriers compared to those in advanced stages (*p* < 0.05).

**Conclusions:**

Colonoscopy is more widely recognized than FOBT among the Turkish adult population; however, a significant proportion remains in the precontemplation stage for screening. Perceptions of benefits and barriers are influenced by sociodemographic factors, health status, and CRC knowledge. Interventions addressing these barriers and raising awareness could improve CRC screening uptake and help reduce the disease burden.

## Introduction

Colorectal cancer (CRC), one of the leading causes of cancer-related mortality and morbidity, is the second most common cancer among women and the third one in men worldwide [1]. In Turkey, CRC occupies third place in women and men with regard to incidence and fifth among the deadliest cancers [2].

Studies have reported that mortality and morbidity rates due to CRC can decrease significantly with screening programs [3, 4]. Turkey’s community-based CRC screening program was launched in 2014, targeting individuals aged 50 to 70. The program includes a fecal occult blood test (FOBT) every 2 years and a colonoscopy every 10 years. However, screening procedures for individuals at high risk may vary based on specific clinical recommendations [2].

Although screening programs are known to reduce CRC-related mortality and morbidity, screening behaviors of risky individuals are not yet at the desired level [5]. In a report published by the American Cancer Society (ACS) in 2020, it was stated that the rate of having a colonoscopy in the USA was 61%, while the rate of having a FOBT was 11% [6]. Similarly, in a report published by the Organisation for Economic Co-operation and Development (OECD), of which Turkey is a member, it was stated that 40.4% of people between the ages of 50 and 74 in OECD member countries had a FOBT at least once in their lives, and that 18.4%, regardless of age, had a colonoscopy at least once in their lives [7]. The Turkey Cancer Control Program report, published by the General Directorate of Public Health in Turkey, states that the coverage rate of CRC screenings is between 30 and 40%, with 24.1% of diagnosed cases being in the advanced stage [2].

Research investigating the reasons behind the low CRC screening rates highlighted several individual and health system-related barriers affecting participation [8–10]. These barriers encompass sociodemographic factors such as age, gender, education level, income, marital status, and employment status, all of which can impact individuals’ awareness, access to resources, and willingness to participate in screening programs [10]. Additional barriers include a lack of knowledge about CRC and its screening methods [8, 11], fear, embarrassment, and anxiety related to screening procedures [8, 9, 11], fatalistic beliefs [9], the perception that testing is unnecessary [8, 9], lack of health insurance [9, 11], and the absence of healthcare provider recommendations for screening tests [10, 11]. Identifying factors that influence participation in CRC screenings is crucial for designing effective interventions aimed at increasing screening behaviors in at-risk individuals [12, 13]. Health behavior theories have been developed to predict why people do or do not engage in health promotion programs [14]. The transtheoretical model (TTM), also known as the Stages of Change (SOC), is among the most frequently utilized frameworks investigating cancer screening behaviors. According to the TTM, an individual’s behavior change is not a result but a process that involves different stages (precontemplation, contemplation, preparation, action, and maintenance), and the individual can move forward or backward between these stages [14–17]. It was suggested that there may be a change in the individual’s perception of barriers and benefits according to the stages of behavior change [14–17].

Although some studies have examined the factors affecting the stages of change in CRC screening behaviors, the number of studies in this field is still limited [16, 17]. Primary healthcare centers are key facilities where preventive health services are provided. Understanding the barriers faced by individuals to undergo CRC screening, as well as assessing their readiness for behavioral change, is expected to guide the development of targeted interventions. Therefore, the study aims to investigate the stages of adoption of CRC screening behaviors, with a specific focus on FOBT and colonoscopy, among adults attending primary healthcare centers. Additionally, the study aims to explore the factors influencing the adoption of CRC screening behaviors, as well as the perceived benefits and barriers associated with CRC screening.

## Methods

### Study design and sample

After obtaining approval from the ethics committee, a cross-sectional survey was conducted in primary healthcare centers across the Anatolian region in Istanbul between May and June 2022. The study utilized a multistage cluster sampling method. Istanbul was first divided into two primary regions, Anatolia and Europe, and the Anatolian region was chosen through random selection. This region, comprising 14 districts, served as the sampling frame. Five districts, Sancaktepe, Maltepe, Kartal, Beykoz, and Üsküdar, were then randomly selected using a simple random sampling approach. A total of 20 primary healthcare centers were selected, with the sample distributed proportionally across districts: 5 centers from Maltepe, 5 from Üsküdar, 4 from Kartal, 3 from Beykoz, and 3 from Sancaktepe. Data was collected from individuals seeking services at these selected centers.

Based on data from the Turkish Statistical Institute, the population aged 50–70 years in Istanbul in 2021 was 2,806,312 [18]. The Colorectal Cancer National Control Plan in Turkey states that the colorectal cancer screening rate among the target Turkish population ranges between 30 and 40% [2]. Using OpenEpi software, the sample size was calculated with an expected frequency of 40%, a 95% confidence interval, and a design effect of 1.5, resulting in a target sample size of 553 participants [19]. A total sample of 498 individuals was recruited, resulting in a participation rate of 90%.

The study included participants aged 50–70 years with no apparent cognitive impairment who provided voluntary informed consent. Individuals with significant cognitive impairment (defined as an SMMT, Turkish version of mini-mental state examination) score < 24 for those aged 65 and older, or a history of CRC diagnosis were excluded.

### Data collection tools

**Fig. 1:**
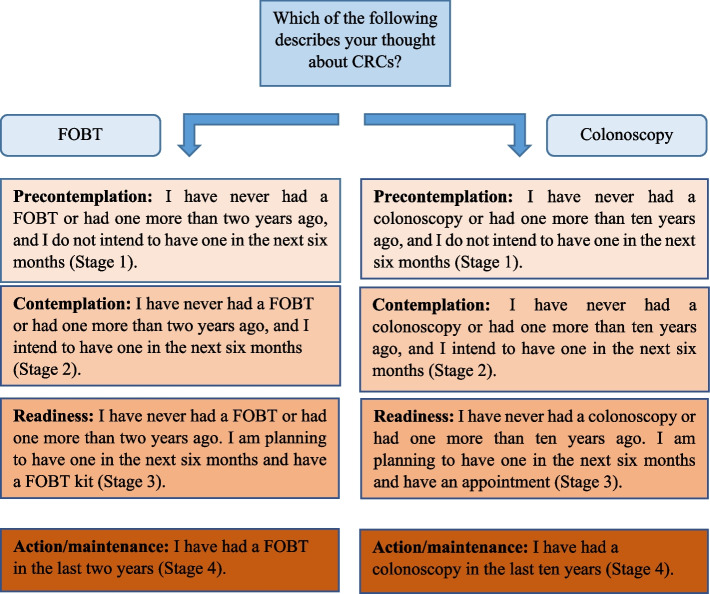
Stages of adoption for FOBT and colonoscopy

The study data were collected using a predesigned structured questionnaire, developed following an extensive literature review [16, 17]. The questionnaire comprised the following sections:

- Sociodemographic characteristics including gender, age, marital status, employment, income. Income status was measured with a single question: “How would you describe your monthly income status?” (response options: good, moderate, poor).
- Family history of CRC and one question about health status: “How would you describe your health status?” (response options: good, moderate, poor).
- Knowledge about CRC screening was evaluated via two questions: “Do you know what a Fecal Occult Blood Test is?” and “Do you know what a colonoscopy is?”.
- CRC screening recommendation by the physician was evaluated with two questions: “Has your doctor ever recommended that you have a FOBT before?” and “Has your doctor ever recommended that you have a colonoscopy before?”.
- Source of information about CRC screening methods was evaluated with two questions: “How did you find out about fecal occult blood testing?” and “How did you find out about a colonoscopy?”.
- The stages of readiness for CRC screening questionnaire were used to assess stages of adoption for CRC screening using transtheoretical model (Fig. 1) [16, 17]. The original version of the questionnaire was translated into Turkish by two Turkish academics who are experts in both Turkish and English. The questions were then back-translated into English by two independent academics from Turkey, following Beaton-recommended guidelines [20]. One statement was designed to describe each stage, and participants were asked to select the stage that best represented their readiness for screening.
- In this study, the “Turkish version of the Instruments to measure CRC screening benefits and barriers” [21] was used to assess perceived benefits and barriers. The scales were originally developed in English by Rawl et al. in 2001 [22]. These instruments specifically evaluate individuals’ perceptions regarding two CRC screening test options: FOBT and colonoscopy. The instruments consist of 31 items evaluating the perceptions about the barriers and benefits of FOBT and colonoscopy. The response to each item in the 4-point Likert-type scale is marked as “strongly disagree” (1 point), “disagree” (2 points), “agree” (3 points), “completely agree” (4 points), “I do not know” (0 point), and “I refuse to answer” (0 point). Before responding to the statements in the scale, the individuals were briefly informed about the FOBT and colonoscopy procedures to facilitate the understanding of the items. The FOBT benefits scale contained three items, and the FOBT barriers scale contained nine items. The colonoscopy benefits scale included 4 items, and the colonoscopy barriers scale consisted of 15 items. In the current study, the Cronbach’s alpha coefficient for the FOBT benefits scale was found to be *α* = 0.87, and for the FOBT barriers scale, it was *α* = 0.71. The Cronbach’s alpha coefficients for the colonoscopy benefits and barriers scales were *α* = 0.87 and *α* = 0.85, respectively.
- The cognitive status of those over 65 years of age was assessed using the Turkish version of the mini-mental state examination [23]. The original version was developed by Folstein in 1975, consists of 11 items, and is evaluated over a total of 30 points. Each question in the test is worth “1” point. The lowest score that can be obtained from the scale is “0,” and the highest score is “30.” Scoring 24 and above on this test indicates that there is no cognitive disorder [24]. Given this benchmark, those over 65 years of age with scores of 24 points or higher were included in the study.
- A pilot test was conducted with 10 participants to evaluate the clarity of all items and their understandability. These participants were not included in the total study sample.

### Statistical analysis

The data were analyzed using the IBM SPSS software, version 21 (IBM, Corp, Armonk, New York). Descriptive data were analyzed using numbers and percentages for categorical variables and mean and standard deviation for continuous variables. The independent samples Student’s *t*-test was employed to compare the means between two groups. In addition, ANOVA (Analysis of Variance) was employed in the comparison of the means of more than two groups, and Tukey and Tamhane’s T2 tests were used as post hoc tests. The statistical significance level was set at *p* < 0.05.

## Results

A total of 498 participants were interviewed for the study. The mean (SD) age of the participants was 59.10 (*SD* = 5.71). More than half were women (54.8%), and 85.7% were married; more than two-thirds (66.1%) had less than a high school education and were not actively employed (67.9%); and most of them perceived their income level and health status as moderate (70.3% and 62.1%, respectively, Table 1).

**Table 1:** General characteristics of the study adults attending primary healthcare centers, Anatolian region, Turkey, 2022 (*n* = 498)

Mean **age** ± *SD* (min–max)	59.10 ± 5.71 (50–70)
**Gender**, *n* (%)	
Male	225 (45.2)
Female	273 (54.8)
**Marital status**, *n* (%)	
Married	427 (85.7)
Single	71 (14.3)
**Educational status**, *n* (%)	
High school and above	169 (33.9)
Below high school	329 (66.1)
**Employment status**, *n* (%)	
Yes	160 (32.1)
No	338 (67.9)
**Income status**, *n* (%)	
Good	103 (20.7)
Moderate	350 (70.3)
Poor	45 (9.0)
**Health status**, *n* (%)	
Good	150 (30.1)
Moderate	309 (62.1)
Poor	39 (7.8)

**Fig. 2:**
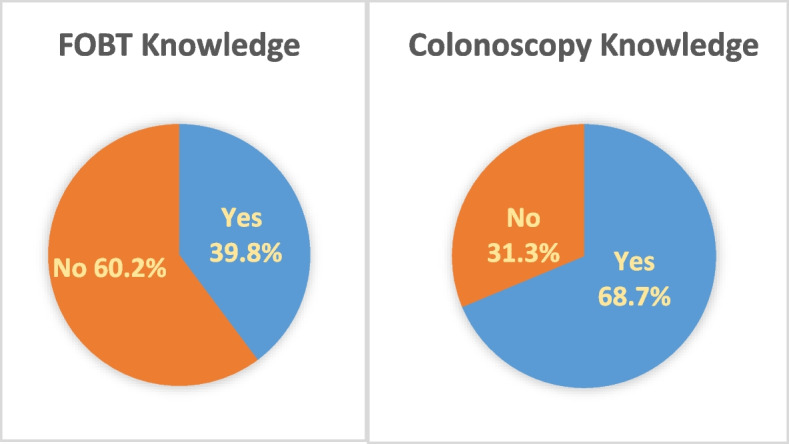
FOBT and colonoscopy knowledge (*n* = 498)

**Fig. 3:**
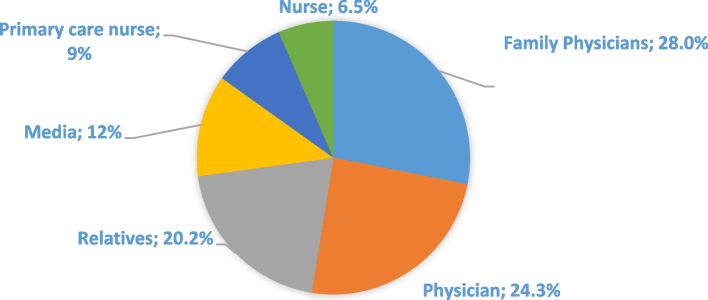
Source of information about FOBT (*n* = 198)

**Fig. 4:**
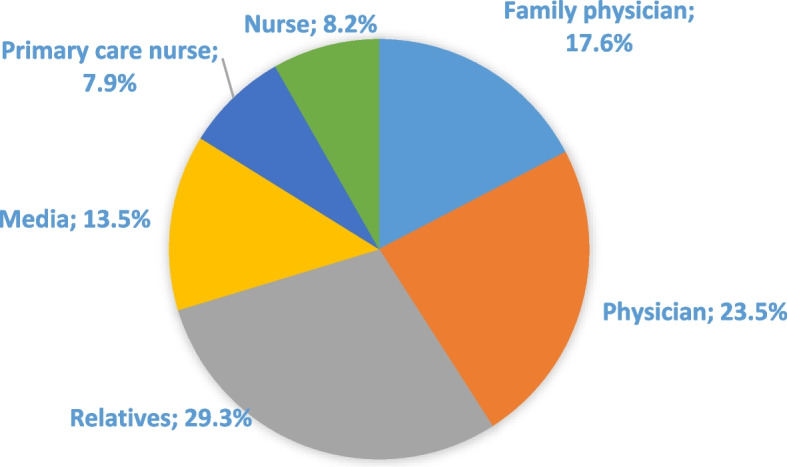
Source of information about colonoscopy (*n* = 342)

The level of knowledge and sources of information regarding CRC screening tests are presented in Fig. 2, Fig. 3, and Fig. 4. The results revealed that 39.8% of participants were informed about FOBT, while 68.7% had knowledge about colonoscopy. It was found that participants primarily received information about FOBT from their family physicians (28%), while information about colonoscopy was mainly obtained from their relatives (29.3%).

**Fig. 5:**
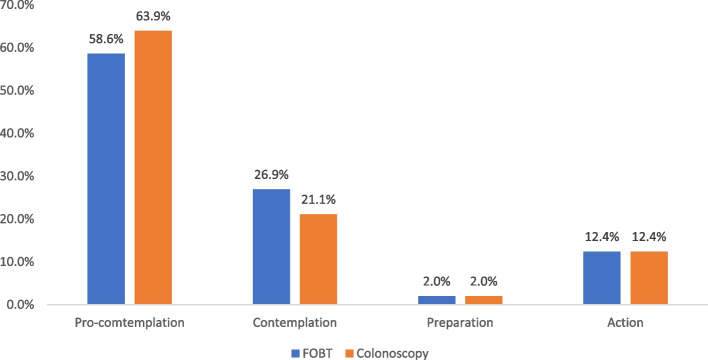
Stages of adoption for FOBT and colonoscopy (*n* = 498)

Among the 498 participants who were included in the analysis of stages of adoption for FOBT, 58.6%, 26.9%, 2%, and 12.4% were categorized into precontemplation, contemplation, readiness, and action/maintenance stages, respectively. Regarding the analysis of the stages of adoption for colonoscopy, 63.9% of the participants were classified in the precontemplation stage, 21.1% were in the contemplation stage, 2% were in the readiness stage, and 12.4% of them were in the action/maintenance stage (Fig. 5).

Table 2 illustrates the factors associated with the adoption of colorectal cancer screening behaviors, specifically focusing on FOBT and colonoscopy. Gender-based variations were less pronounced, with the male and female participants showing similar rates of stages of adoption through FOBT and colonoscopy (*p* > 0.05). Marital status revealed significant differences in FOBT stage of adoption, with those married individuals more likely to be at Stage 1 (60.4%) compared to the single individuals (47.9%, *p* = 0.043). However, no significant difference was observed in colonoscopy results (*p* = 0.481). Educational status also influenced screening behaviors. Among the individuals with education below high school, 62.6% were at Stage 1 through FOBT, compared to 50.9% of those with a high school education or higher (*p* = 0.017). Similarly, for colonoscopy, 68.4% of the participants with lower education were at Stage 1, compared to 55.0% of those with higher education (*p* = 0.014).

**Table 2:** Factors associated with the adoption of colorectal cancer screening behaviors (*n* = 498)

Factors	**FOBT**				**Colonoscopy**				
	**Stage1** ***n* (%)**	**Stage2** ***n* (%)**	**Stage3** ***n* (%)**	**Stage4** ***n* (%)**	**Stage1** ***n* (%)**		**Stage2** ***n* (%)**	**Stage3** ***n* (%)**	**Stage4** ***n* (%)**
**Gender**									
Male	135 (60.0)	54 (24.0)	5 (2.2)	31 (13.8)	134 (59.6)		47 (20.9)	6 (2.7)	38 (16.8)
Female	157 (57.5)	80 (29.3)	5 (1.8)	31 (11.4)	184 (67.4)		58 (21.2)	4 (1.5)	27 (9.9)
* p*-value	0.553				0.082				
**Marital status**									
Married	258 (60.5)	106 (24.8)	10 (2.3)	53 (12.4)	277 (64.9)		89 (20.8)	9 (2.1)	52 (12.2)
Single	34 (47.9)	28 (39.4)	-	9 (12.7)	41 (57.8)		16 (22.5)	1 (1.4)	13 (18.3)
* p*-value	0.043*				0.481				
**Educational status**									
High school and above	86 (50.9)	48 (28.4)	4 (2.4)	31 (18.3)	93 (55.0)		40 (23.7)	5 (3.0)	31 (18.3)
Below high school	206 (62.6)	86 (26.1)	6 (1.8)	31 (9.5)	225 (68.4)		65 (19.8)	5 (1.5)	34 (10.3)
* p*-value	0.017*				0.014*				
**Employment status**									
Yes	80 (50.0)	49 (30.6)	4 (2.5)	27 (16.9)	94 (58.7)		37 (23.1)	6 (3.8)	23 (14.4)
No	212 (62.7)	85 (25.1)	6 (1.8)	35 (10.4)	224 (66.3)		68 (20.1)	4 (1.2)	42 (12.4)
* p*-value	0.042*				0.151				
**Income status**									
Good	41 (39.8)	35 (34.0)	5 (4.9)	22 (21.3)	53 (51.4)		23 (22.3)	5 (4.9)	22 (21.4)
Moderate	221 (63.1)	87 (24.9)	5 (1.4)	37 (10.6)	228 (65.1)		75 (21.4)	5 (1.4)	42 (12.1)
Poor	30 (66.7)	12 (26.7)	-	3 (6.6)	37 (82.2)		7 (15.6)	-	1 (2.2)
* p*-value	< 0.001*				0.002*				
**Health status**									
Good	79 (52.7)	41 (27.3)	5 (3.3)	25 (16.7)	95 (63.3)		31 (20.7)	5 (3.3)	19 (12.7)
Moderate	187 (60.5)	85 (27.5)	4 (1.3)	33 (10.7)	200 (64.7)		65 (21.0)	5 (1.6)	39 (12.6)
Poor	26 (66.6)	8 (20.5)	1 (2.6)	4 (10.3)	23 (59.0)		9 (23.1)	-	7 (17.9)
* p*-value	0.286				0.760				
**Family history of colorectal cancer**									
Yes	21 (43.8)	14 (29.3)	4.2 (2.0)	11 (22.9)	20 (41.7)		9 (18.8)	1 (2.1)	18 (37.4)
No	271 (60.2)	120 (26.7)	8 (1.8)	51 (11.3)	298 (66.2)		96 (21.4)	9 (2.0)	47 (10.4)
* p*-value	0.046*				< 0.001*				
**Colorectal cancer screening recommendation**									
Yes	30 (25.0)	36 (30.0)	9 (7.5)	45 (37.5)	33 (27.5)		32 (26.7)	6 (5.0)	49 (40.8)
No	262 (69.3)	98 (25.9)	1 (0.3)	17 (4.5)	282 (75.4)		73 (19.3)	4 (1.1)	16 (4.2)
* p*-value	< 0.001*				< 0.001*				
**FOBT knowledge**									
Yes	65 (32.8)	65 (32.8)	9 (4.5)	59 (29.8)	-				
No	227 (75.7)	69 (23.0)	1 (0.3)	3 (1.0)	-				
* p*-value	< 0.001*				-				
**Colonoscopy knowledge**									
Yes	-				190 (55.6)		81 (23.7)	8 (2.3)	63 (18.4)
No	-				128 (82.1)		24 (15.4)	2 (1.3)	2 (1.3)
* p*-value	-				< 0.001*				

^*^p < 0.05 (significant)

Employment status showed significant differences in FOBT stages of adoption, with the unemployed participants more likely to be at Stage 1 compared to the employed participants (*p* = 0.042). However, for colonoscopy, no statistically significant difference was observed between the employed and unemployed participants (*p* = 0.151). Income status was significantly associated with the stage of adoption. The participants with good income had the highest rates of Stages 2, 3, and 4 rates for FOBT and the highest Stages 3 and 4 rates for colonoscopy (*p* < 0.001). Those participants with a family history of colorectal cancer were less likely to be at Stage 1 compared to those without a family history for both FOBT and colonoscopy (*p* = 0.046 and < 0.001, respectively).

Screening recommendations and knowledge were strongly associated with diagnostic stages. The participants who received screening recommendations had substantially lower Stage 1 diagnosis rates compared to those without recommendations colonoscopy (*p* < 0.001). Similarly, those with knowledge of FOBT and colonoscopy had lower Stage 1 diagnosis rates than those without such knowledge (*p* < 0.001).

Perceived benefits of FOBT or colonoscopy were significantly higher among participants with higher education (*p* < 0.001), employment (*p* < 0.001 for FOBT and *p* = 0.046 for colonoscopy), income levels (*p* < 0.001), as well as those with CRC knowledge (*p* < 0.001), and screening recommendations from healthcare providers (*p* < 0.001). Conversely, barriers were notably lower in participants with prior knowledge about FOBT (*p* < 0.001) or colonoscopy (*p* = 0.043) and those receiving screening recommendations (*p* = 0.028, Table 3).

**Table 3:** Factors associated with participants’ perceived benefits and barriers to FOBT and colonoscopy (*n* = 498)

Factors	FOBT		Colonoscopy	
	**Benefits**	**Barriers**	**Benefits**	**Barriers**
**Gender**				
Male	2.52 ± 1.41	2.20 ± 0.64	2.68 ± 0.98	2.25 ± 0.62
Female	2.38 ± 1.41	2.21 ± 0.68	2.53 ± 1.08	2.21 ± 0.68
* p*-value	0.281	0.922	0.097	0.500
**Marital status**				
Married	2.42 ± 1.42	2.20 ± 0.68	2.57 ± 1.03	2.23 ± 0.66
Single	2.61 ± 1.32	2.22 ± 0.54	2.79 ± 1.05	2.20 ± 0.61
* p*-value	0.273	0.857	0.095	0.716
**Educational status**				
High school and above	2.95 ± 1.12	2.14 ± 0.59	2.91 ± 0.82	2.15 ± 0.66
Below high school	2.18 ± 1.47	2.24 ± 0.69	2.44 ± 1.10	2.26 ± 0.65
* p*-value	** < 0.001**	0.090	** < 0.001**	0.082
**Employment status**				
Yes	2.78 ± 1.29	2.16 ± 0.65	2.73 ± 1.00	2.15 ± 0.66
No	2.28 ± 1.44	2.23 ± 0.66	2.53 ± 1.05	2.26 ± 0.65
* p*-value	** < 0.001**	0.327	**0.046**	0.095
**Income status**				
Good	3.10 ± 1.07*	2.16 ± 0.57	2.97 ± 0.84*	2.15 ± 0.60
Moderate	2.31 ± 1.42	2.24 ± 0.67	2.51 ± 1.06	2.24 ± 0.66
Poor	2.00 ± 1.59	2.08 ± 0.80	2.40 ± 1.08	2.34 ± 0.70
* p*-value	** < 0.001**	0.242	** < 0.001**	0.252
**Health status**				
Good	2.71 ± 1.29*	2.18 ± 0.73	2.79 ± 0.92*	2.25 ± 0.69
Moderate	2.36 ± 1.43	2.22 ± 0.63	2.51 ± 1.08	2.21 ± 0.65
Poor	2.07 ± 1.54	2.21 ± 0.59	2.52 ± 1.06	2.25 ± 0.56
* p*-value	**0.030**	0.892	**0.015**	0.855
**Family history of colorectal cancer**				
Yes	3.01 ± 0.91	2.24 ± 0.57	2.96 ± 0.60	2.26 ± 0.56
No	2.38 ± 1.44	2.20 ± 0.67	2.56 ± 1.07	2.22 ± 0.66
* p*-value	** < 0.001**	0.723	** < 0.001**	0.678
**Colorectal cancer screening recommendation**				
Yes	3.03 ± 1.02	2.09 ± 0.58	2.90 ± 0.86	2.14 ± 0.60
No	2.26 ± 1.47	2.24 ± 0.68	2.50 ± 1.07	2.25 ± 0.67
* p*-value	** < 0.001**	**0.028**	** < 0.001**	0.102
**FOBT knowledge**				
Yes	3.24 ± 0.75	2.08 ± 0.55	2.99 ± 0.77	2.10 ± 0.59
No	1.92 ± 1.50	2.29 ± 0.71	2.34 ± 1.11	2.31 ± 0.68
* p*-value	** < 0.001**	** < 0.001**	** < 0.001**	** < 0.001**
**Colonoscopy knowledge**				
Yes	2.74 ± 1.26	2.16 ± 0.64	2.87 ± 0.81	2.23 ± 0.62
No	1.79 ± 1.51	2.29 ± 0.69	2.00 ± 1.22	2.22 ± 0.74
* p*-value	** < 0.001**	**0.043**	** < 0.001**	0.954

^*^p < 0.05 (significant)

When the stages of change in CRC screening behavior and the mean scores of CRC screening (FOBT and colonoscopy) benefit perceptions were compared, the results of Table 3 show that the mean scores of the perception of benefits of the individuals in the precontemplation stage were lower than those of the individuals in the upper stages (contemplation, preparation, and action), and that the difference between them was statistically significant (*p* < 0.001). In addition, the comparison of CRC screening stages of adoption and the mean scores of barriers perception regarding CRC screening (FOBT and colonoscopy) revealed that the mean scores of barriers perception of the individuals in the precontemplation stage were higher than the mean scores of the individuals in the upper stages (contemplation, preparation, and action), and that the difference between them was also statistically significant (*p* = 0.017 for FOBT and *p* = 0.046 for colonoscopy, Table 4).

**Table 4:** Perceived benefits and barriers across stages of CRC screening behavior change

Stages	FOBT		Colonoscopy	
	**Benefits**	**Barriers**	**Benefits**	**Barriers**
TTM phase				
Precontemplation	2.03 ± 1.48*	2.31 ± 0.71*	2.39 ± 1.11*	2.31 ± 0.67*
Contemplation	2.85 ± 1.16	2.11 ± 0.55	2.91 ± 0.82	2.12 ± 0.64
Preparation	3.60 ± 0.46	2.03 ± 0.46	2.88 ± 0.78	2.08 ± 0.66
Action	3.32 ± 0.75	1.94 ± 0.58	3.08 ± 0.69	1.99 ± 0.50
*p*-value	** < 0.001**	**0.017**	** < 0.001**	**0.046**

^*^p < 0.05 (significant)

## Discussion

This study aimed to investigate CRC screening behaviors among individuals aged 50–70 according to the stages of behavior change and to examine factors influencing the adoption of CRC screening behaviors, as well as the perceived benefits and barriers associated with CRC screening.

The analysis of CRC change stages of the screening behavior revealed that most participants in the study were in the precontemplation stage for both FOBT (58.6%) and colonoscopy (63.9%). In contrast, in Korea, Bui et al. [25] reported significantly lower rates of precontemplation (*FOBT*: 17.7%, colonoscopy: 20.6%), with a higher proportion of individuals in the contemplation and action stages [25]. This discrepancy may be attributed to differences in cultural, social, and healthcare system factors. Limited awareness, insufficient public health campaigns, lower perceived susceptibility to CRC, and systemic barriers such as cost and availability of screening services may contribute to the higher precontemplation rates observed in this study.

Additionally, the findings indicate that individuals with higher perceived benefits of CRC screening were more likely to progress toward the action stage, while those with greater perceived barriers remained in the precontemplation stage. These results align with other studies where the majority of participants were also in the precontemplation stage [26, 27]. However, a study conducted in Singapore found that most participants were in the preparation and maintenance stages [28], suggesting that readiness for behavior change plays a critical role in screening participation [29]. These findings underscore the importance of targeted public health strategies to address specific barriers and enhance CRC screening uptake.

In the present study, 39.8% of participants had knowledge about FOBT, while 68.7% were aware of colonoscopy. The primary sources of information were family physicians for FOBT and relatives for colonoscopy. These findings align with Oh et al. [30] results among Korean Americans, where 71% of participants had heard about FOBT and 84% about colonoscopy [30]. However, Xu et al. [31] in China reported that 84.9% of CRC patients had no prior knowledge of screenings before diagnosis, with their main sources of information being family and friends with cancer, social media, television, and community education [31].

This study, along with previous research, reinforces that colonoscopy is the most widely recognized CRC screening method, as it is considered the gold standard [32]. However, concerns about preparation, pain, discomfort, and embarrassment often lead to negative perceptions, discouraging participation [33]. This highlights the importance of FOBT, especially in primary healthcare settings, where physicians and nurses play a crucial role in educating individuals and promoting screening participation. In Turkey, family physicians are the primary source of information on FOBT, which is offered free of charge at family health centers and recommended during consultations. In contrast, colonoscopy is performed in secondary healthcare facilities, such as hospitals or Cancer Early Diagnosis, Screening, and Training Centers (KETEM).

A notable finding in this study is that most participants received information about colonoscopy from their social circles rather than healthcare professionals, suggesting that primary healthcare providers may not be offering sufficient counseling on CRC screening. Addressing this gap through improved communication and education by primary care physicians could enhance screening awareness and participation. Further research is needed to explore strategies for strengthening the role of healthcare providers in CRC screening education.

In this study, marital status, education, employment, income, and family history of colorectal cancer were identified as factors related to the stages of adoption for FOBT and colonoscopy. Wang et al. [16] in their study among Hispanics in the USA also found significant differences between age, gender, cancer history, and provider recommendations in the stage of adoption for FOBT and colonoscopy [34]. We observed that gender was not related to the stages of adoption for FOBT and colonoscopy. Unlike our study, in Ethiopia, Hamza et al. [35] found that women were 1.86 times more aware than men about screening [35]. Likewise, it was reported in a systematic review that women participated in screenings more frequently than men [36]. This discrepancy could be due to differences in health-seeking behaviors, social support, or targeted health promotion campaigns in the studied populations. Additionally, differences in the healthcare systems, such as the availability of screening programs or provider outreach, may contribute to these contrasting findings. For example, in settings where women have more access to preventive healthcare or are specifically targeted in public health campaigns, their participation rates and awareness levels are likely to be higher. These factors underscore the need to consider contextual and systemic influences when interpreting and comparing findings across studies.

In this study, marital status was found to be related to the stages of adoption for FOBT, with married participants more likely to engage in screening. This finding is consistent with previous research, which has shown that married individuals tend to participate in screenings more frequently than single ones [36, 37]. Furthermore, participants with higher educational status, employment, higher income, knowledge about FOBT, a family history of colorectal cancer (CRC), better self-assessed health status, and those who received screening recommendations from healthcare providers had significantly higher perceived benefit scores. These results align with existing literature, which reports that individuals with higher educational attainment [35, 36], higher income levels [35, 36], better self-perceived health status [36, 38], and employment [36, 38], as well as those with a family history of CRC, are more likely to agree to screening and have more positive perceptions of its benefits [35, 36]. These factors can be considered reinforcing factors that enhance participation in screening.

In this study, participants who received screening recommendations and had information about screening tests reported significantly lower barrier perception scores. This aligns with the findings from Hatamian et al. [39], who identified key facilitators for CRC screening, including knowledge and awareness about screenings, having a family history of cancer, and receiving a doctor’s recommendation [39]. In addition, a review of qualitative studies highlighted several barriers that hinder first-degree relatives’ (FDRs) compliance with colonoscopy recommendations. These barriers included negative attitudes toward screening (e.g., fear of pain, discomfort, and embarrassment), fear of abnormal test results or a cancer diagnosis, high procedure costs, limited healthcare access, lack of awareness about increased CRC risk, an external locus of control, and time constraints related to preparation and screening processes [40]. In this context, family physicians and nurses in primary healthcare play crucial roles. Nurses, in particular, are integral to both primary and secondary prevention. Their primary prevention responsibilities include educating individuals about CRC risk factors, weight control, exercise, diet, and overall wellness. In secondary prevention, nurses provide information about screenings and organize navigation programs to increase participation [29].

### Limitations of the study

Although this study provides valuable insights, several limitations should be considered when interpreting the results. First, the cross-sectional design prevents the establishment of causal relationships between factors influencing CRC screening behaviors and the stages of adoption. Second, the reliance on self-reported data introduces the potential for recall bias or social desirability bias. Lastly, the study did not examine additional psychosocial factors, such as fear or stigma, which may also impact CRC screening behaviors. Future research should address these limitations by employing longitudinal designs, larger and more diverse samples, and a more comprehensive assessment of influencing factors.

## Conclusions

The study revealed that colonoscopy is more widely recognized than FOBT, with most participants receiving information about colonoscopy from relatives. The majority of participants were in the precontemplation stage for both FOBT and colonoscopy. Factors influencing the stages of behavior change included marital status, education, employment, income, family history of colorectal cancer, provider recommendation, and knowledge of screening tests. Perceptions of benefits and barriers were also significant predictors of screening behaviors.

Nurses in primary care should evaluate individuals’ readiness for CRC screening using the transtheoretical model and implement stage-specific interventions. Personalized and culturally sensitive education and motivational interviewing techniques are recommended to enhance screening uptake. Public health strategies should integrate community-wide, culturally tailored educational programs that align with local traditions, values, and needs. Increasing public awareness and implementing evidence-based initiatives to address screening barriers and benefits can improve participation rates and contribute to the program’s long-term effectiveness.

## Data Availability

Data is available from the corresponding author upon reasonable request.

## Abbreviations

ACS: American Cancer Society
CRC: Colorectal cancer
FOBT: Fecal occult blood test
FHC: Family Health Center
KETEM: Cancer Early Diagnosis, Screening, and Training Center
OECD: Organisation for Economic Co-operation and Development
ORCID: Open Researcher and Contributor ID
SD: Standard deviation
SMMT: Standardized Mini-Mental Test
SOC: Stages of change
SPSS: Statistical Package for the Social Sciences
TTM: Transtheoretical model
WHO: World Health Organization
